# Supplementary material for: Age, Rather Than Social Vulnerability, Is Associated With Disparities in Prescription Patterns in Inflammatory Bowel Disease: An Epic Cosmos Study
Source: Gastro Hep Adv. 2026 Jul 31;5(11):101078. doi: 10.1016/j.gastha.2026.101078 (PMC13546900; doi:10.1016/j.gastha.2026.101078)
Supplement: Supplementary file 1 [file mmc1.pdf]

Supplemental Table 1: List of RxNorm Codes Used for Identification of Medication Classes.

Medication Class	Medications	RxNorm
Anti-TNF Biologics	Adalimumab	327361
	Certolizumab	709271
	Golimumab	819300
	Infliximab	191831
Anti-Integrin Biologics	Natalizumab	354770
	Vedolizumab	1538097
Anti-IL-12/23 Biologics	Ustekinumab	847083
	Risankizumab	2166040
	Mirikizumab	2668384
Immunomodulators	Azathioprine	1256
	Cyclosporine	3008
	Mercaptopurine	103
	Methotrexate	6851
	Tacrolimus	42316
5-ASAs	Balsalazide	18747
	Mesalamine	52582
	Sulfasalazine	9524
JAK Inhibitors	Tofacitinib	1357536
	Upadacitinib	2196092
S1P Modulators	Ozanimod	2288236
	Etrasimod	2668045
Corticosteroids	Budesonide	19831
	Methylprednisolone	6902
	Prednisone	8640

Abbreviations: TNF - Tumor Necrosis Factor, IL - Interleukin, ASA - Aminosalicylic Acid, JAK - Janus Kinase, S1P - Sphingosine 1-phosphate

Supplemental Table 2: IBD Medication Exposure Classified by Female Age Group (Reference Group is Age 18-25 years old).

**Medication Class**	**Age Group (Years)**	**Odds Ratio**	**Lower 95% CI**	**Upper 95% CI**	***P*-value**
Anti-TNF Biologics	26-45	0.76	0.64	0.89	**< .001**
	46-65	0.46	0.39	0.55	**< .001**
	65+	0.16	0.12	0.21	**< .001**
Anti-Integrin	26-45	1.06	0.64	1.77	.822
	46-65	0.81	0.48	1.37	.415
	65+	0.62	0.30	1.25	.190
Anti-IL-12/23	26-45	0.91	0.71	1.17	.469
	46-65	0.61	0.46	0.79	**< .001**
	65+	0.25	0.16	0.38	**< .001**
Immunomodulators	26-45	0.98	0.82	1.19	.866
	46-65	1.09	0.91	1.30	.357
	65+	1.04	0.84	1.29	.741
5-ASA	26-45	0.91	0.80	1.03	.125
	46-65	0.90	0.79	1.01	.077
	65+	1.30	1.13	1.50	**< .001**
JAK Inhibitors	26-45	0.79	0.40	1.57	.494
	46-65	1.08	0.59	2.07	.803
	65+	0.28	0.09	0.76	**.017**
Corticosteroids	26-45	1.19	1.08	1.32	**.001**
	46-65	1.40	1.27	1.55	**< .001**
	65+	1.48	1.30	1.67	**< .001**

Abbreviations: TNF - Tumor Necrosis Factor, IL - Interleukin, ASA - Aminosalicylic Acid, JAK - Janus Kinase

Supplemental Table 3: IBD Medication Exposure Classified by Insurance Status.

Medication Class	Exposed n (%)	Insurance Status	Odds Ratio	Lower 95% CI	Upper 95% CI	*P*-value
Anti-TNF Biologics	2,095 (3.48%)	Medicaid (n= 225)	Reference	–	–	–
		Medicare (n= 369)	0.95	0.80	1.12	.528
		Miscellaneous/Other (n=1,372)	1.09	0.94	1.26	.258
		Self-Pay (n= 129)	0.96	0.76	1.19	.693
Anti-Integrin Biologics	213 (0.35%)	Medicaid (n= 16)	Reference	–	–	–
		Medicare (n= 47)	1.56	1.10	2.22	.128
		Miscellaneous/Other (n=132)	1.44	0.88	2.53	.168
		Self-Pay (n= 18)	1.93	1.12	3.31	.057
Anti-IL-12/23 Biologics	712 (1.18%)	Medicaid (n= 75)	Reference	–	–	–
		Medicare (n= 157)	1.20	0.91	1.60	.199
		Miscellaneous/Other (n=433)	1.05	0.82	1.35	.717
		Self-Pay (n=47)	1.07	0.73	1.54	.727
Immunomodulators	2,123 (3.53%)	Medicaid (n= 239)	Reference	–	–	–
		Medicare (n= 359)	0.81	0.69	0.96	**.016**
		Miscellaneous/Other (n=1,363)	1.00	0.87	1.16	.99
		Self-Pay (n=162)	1.15	0.94	1.41	.184
5-ASAs	5,843 (9.71%)	Medicaid (n= 690)	Reference	–	–	–
		Medicare (n= 1079)	0.83	0.75	0.92	**< .001**
		Miscellaneous/Other (n=3,678)	0.92	0.84	1.00	.062
		Self-Pay (n= 396)	0.96	0.84	1.09	.523
JAK Inhibitors	160 (0.27%)	Medicaid (n= 16)	Reference	–	–	–
		Medicare (n=33)	1.18	0.65	2.21	.593
		Miscellaneous/Other (n=98)	1.11	0.67	1.56	.71
		Self-Pay (n=13)	1.36	0.64	2.10	.413
Corticosteroids	15,083 (25.06%)	Medicaid (n= 1621)	Reference	–	–	–
		Medicare (n= 2823)	0.94	0.87	1.00	.066
		Miscellaneous/Other (n=9,611)	1.06	0.99	1.12	.073
		Self-Pay (n=1,028)	1.09	0.99	1.19	.067

Abbreviations: IBD - Inflammatory Bowel Disease, SVI - Social Vulnerability Index, CI - Confidence Interval, TNF - Tumor Necrosis Factor, IL - Interleukin, ASA - Aminosalicylic Acid, JAK - Janus Kinase

Supplemental Table 4. IBD Medication Exposure Classified by Sex.

Medication Class	Exposed n (%)	Sex	Odds Ratio	Lower 95% CI	Upper 95% CI	*P*-value
Anti-TNF Biologics	2,095 (3.48%)	Men (n=933)	1.06	0.97	1.16	.17
		Women (n=1,162)	-	-	-	-
Anti-Integrin Biologics	213 (0.35%)	Men (n=82)	0.83	0.63	1.10	.20
		Women (n=131)	-	-	-	-
Anti-IL-12/23 Biologics	712 (1.18%)	Men (n=298)	0.96	0.83	1.12	.62
		Women (n=414)	-	-	-	-
Immunomodulators	2,123 (3.53%)	Men (n=903)	0.98	0.90	1.07	.66
		Women (n=1,220)	-	-	-	-
5-ASAs	5,843 (9.71%)	Men (n=2,496)	0.99	0.94	1.05	.85
		Women (n=3,347)	-	-	-	-
JAK Inhibitors	160 (0.27%)	Men (n=69)	1.01	0.74	1.38	.96
		Women (n=91)	-	-	-	-
Corticosteroids	15,083 (25.06%)	Men (n=6,553)	1.03	0.99	1.07	.13
		Women (n=8,530)	-	-	-	-

Abbreviations: IBD - Inflammatory Bowel Disease, SVI - Social Vulnerability Index, CI - Confidence Interval, TNF - Tumor Necrosis Factor, IL - Interleukin, ASA - Aminosalicylic Acid, JAK - Janus Kinase

Supplemental Table 5: IBD Medication Exposure Based on Rural-Urban Differences.

Medication Class	Exposed n (%)	RUCA (n)	Odds Ratio	Lower 95% CI	Upper 95% CI	*P*-value
Anti-TNF Biologics	2,095 (3.48%)	Rural (n= 128)	Reference	–	–	–
		Urban (n= 1967)	1.05	0.87	1.26	.632
Anti-Integrin Biologics	213 (0.35%)	Rural (n= 16)	Reference	–	–	–
		Urban (n=	0.89	0.55	1.54	.649

		197)				
Anti-IL-12/23 Biologics	712 (1.18%)	Rural (n= 40)	Reference	–	–	–
		Urban (n= 672)	1.18	0.87	1.66	.308
Immunomodulators	2,123 (3.53%)	Rural (n= 155)	Reference	–	–	–
		Urban (n= 1968)	0.88	0.74	1.04	.123
5-ASAs	5,843 (9.71%)	Rural (n= 365)	Reference	–	–	–
		Urban (n=5478)	1.07	0.96	1.20	.243
JAK Inhibitors	160 (0.27%)	Rural (n= 12)	Reference	–	–	–
		Urban (n= 148)	0.86	0.49	1.64	.608
Corticosteroids	15,083 (25.06%)	Rural (n= 1001)	Reference	–	–	–
		Urban (n= 14082)	0.98	0.91	1.06	.593

Abbreviations: IBD - Inflammatory Bowel Disease, SVI - Social Vulnerability Index, CI - Confidence Interval, TNF - Tumor Necrosis Factor, IL - Interleukin, ASA - Aminosalicylic Acid, JAK - Janus Kinase
