# Supplementary material for: Age, Rather Than Social Vulnerability, Is Associated With Disparities in Prescription Patterns in Inflammatory Bowel Disease: An Epic Cosmos Study
Source: Gastro Hep Adv. 2026 Jul 31;5(11):101078. doi: 10.1016/j.gastha.2026.101078 (PMC13546900; doi:10.1016/j.gastha.2026.101078)
Supplement: Supplementary file 2 [file mmc2.pdf]

Gastro Hep Advances 2026;5:101078

ORIGINAL RESEARCH—CLINICAL

Age, Rather Than Social Vulnerability, Is Associated With Disparities in Prescription Patterns in Inflammatory Bowel Disease: An Epic Cosmos Study

Hannah Zuercher,[1] Tomas Potlach,[1] Mousumi Sinha,[2] Naveen Baskaran,[1] Shay S. Bidani,[1] Sean Olson,[1] Tianze Jiao,[3] Ellen M. Zimmermann,[1] and Naueen A. Chaudhry[1]

[1]Department of Internal Medicine, Division of Gastroenterology and Hepatology, University of Florida, Gainesville, Florida; [2]Health Science Center, University of Texas, Houston, Texas; and [3]College of Pharmacy, Department of Pharmaceutical Outcomes & Policy, University of Florida, Gainesville, Florida

BACKGROUND AND AIMS: Advanced therapies have transformed the management of inflammatory bowel disease (IBD), yet disparities in their use remain poorly characterized. This study aims to describe nationwide patterns of IBD medication exposure and examine associations with demographic, social, and geographic factors using a newly available, large electronic health records based database. **METHODS:** A retrospective study was conducted using Epic Cosmos, an electronic health record database representing 300 million lives. Patients with more than 1 International Classification of Diseases, Tenth Revision, Clinical Modification diagnosis code for Crohn's disease or ulcerative colitis between 2015 and 2024 were included. Descriptive statistics and demographic distribution were studied for all variables. Multivariate regression compared the exposure odds of IBD-specific medications. **RESULTS:** Our study included 60,187 patients that met criteria. Overall, 43.60% of patients with IBD were exposed to advanced therapies over the timeframe studied. Consistent with prior literature, patients >25 years were less likely to receive anti–interleukin-23/23 biologics, immunomodulators, and Janus kinase inhibitors. Medicare patients were less likely to receive immunomodulators (odds ratio 0.81, 95% confidence interval [0.69, 0.96], $P = .016$) and 5-aminosalicylic acids (odds ratio 0.83, 95% confidence interval [0.75, 0.92], $P < .001$). Few racial and ethnic differences in drug exposure were noted. Small racial and ethnic differences in IBD medication usage were demonstrated. Social Vulnerability Index, urban-rural differences, and sex did not demonstrate significant disparities in medication use. **CONCLUSION:** To our knowledge, this is the first study to assess IBD care in Epic Cosmos. Advanced IBD therapies were less likely to be prescribed to older adults. Social Vulnerability Index, sex, and urban-rural differences were not strong predictors of advanced IBD therapy use, highlighting the complexity of SDOH in IBD care.

Keywords: Race/Racial; Inflammatory Bowel Disease; Equity; Sex; Disparities

Introduction

Modern care pathways for patients with Crohn's disease (CD) and ulcerative colitis (UC) include the rapidly expanding group of FDA-approved advanced therapies, such as biologics and small molecules, which are immune pathway inhibitors. These remarkable therapies have revolutionized our approach to patient care and have improved the quality of life and disease outcomes for millions of patients worldwide.[1–4] How variables such as age, sex, and race impact drug exposure and disease outcomes is an intense area of study with much to learn. On a population basis, social determinants of health (SDOH) have been recognized as critical factors that influence outcomes of many chronic diseases, including inflammatory bowel disease (IBD).[5–7] The Social Vulnerability Index (SVI) is a US census-tract-derived metric from the Centers for Disease Control and Prevention that measures the vulnerability of a community based on demographic and socioeconomic factors, such as housing, transportation, racial/ethnic minority status, health insurance status, and household composition.[8] The association between patient demographics and access to advanced therapies for IBD remains unclear. Research in this area is hampered by dataset size and geographic limitations, as well as timeliness of the available data in this rapidly expanding therapeutic landscape.

Epic Cosmos is an electronic health record (EHR) database comprised of deidentified patient data (from the Epic EHR), representing 300 million lives who were treated in 1809 hospitals and more than 41 thousand clinics in the United States.[9,10] The patient population in Epic Cosmos reflects the US Census population.[9] Epic Cosmos has been utilized in other therapeutic areas, including ophthalmology,

Abbreviations used in this paper: **ASA, aminosalicylic acid; CI, confidence interval; CD, Crohn's disease; EHR, electronic health record; IBD, inflammatory bowel disease; ICD, International Classification of Diseases; IL, interleukin; JAK, Janus kinase; OR, odds ratio; RUCA, Rural-Urban Commuting Area; SDOH, social determinants of health; SVI, Social Vulnerability Index; TNF, tumor necrosis factor.**

Most current article

© 2026 The Authors. Published by Elsevier Inc. on behalf of American Gastroenterological Association Institute. This is an open access article under the CC BY license (http://creativecommons.org/licenses/by/4.0/).
2772-5723
https://doi.org/10.1016/j.gastha.2026.101078

psychiatry, and pediatrics.[9,11,12] However, to our knowledge, no published studies have used Epic Cosmos to investigate the IBD population. Using this nationwide database, this study focuses on SVIs and urban-rural differences, to investigate potential health-care disparities with respect to advanced IBD therapy exposure. We hypothesized that female sex, non-White race, Hispanic or Latino ethnicity, lower socioeconomic status, and higher SVI would be risk factors for a lower probability of receiving advanced IBD therapies. Given the importance of early IBD disease management on patients' long-term quality of life and disease progression, it is vital to increase awareness of the disparities and related factors to mitigate their impacts on disease outcomes.

Materials and Methods

A retrospective study was conducted using the Epic Cosmos database that included patients from October 1, 2015, to October 1, 2024. The date of October 1, 2015 was chosen based on the earliest SVI data available and when the International Classification of Diseases, Tenth Revision, Clinical Modification (ICD-10-CM) code was implemented in the United States. Data used in this study came from Epic Cosmos, a dataset created in collaboration with a community of Epic health systems representing more than 300 million patient records from over 1809 hospitals and over 41,000 clinics from all 50 states.

Patients entered the study cohort at their second visit with an ICD-10 CM diagnosis code for CD or UC (ICD-10 codes: K50 and K51). This visit date was defined as the index date. Visit types, such as emergency department visits, hospitalizations, or outpatient clinic visits, for such diagnosis codes were neither differentiated between nor was the cohort restricted to patients solely seen by a gastroenterologist as the visit provider. Patients aged <18 years at index date or those with other common autoimmune comorbidities including lupus, rheumatoid arthritis, and psoriasis before the index date were excluded (Figure 1). We extracted patients' demographics (age, sex, race, ethnicity, insurance status), SVI scores, and Rural-Urban Commuting Area (RUCA) codes at the patient's baseline, within 1 year prior to the index date. RUCA codes are a classification system developed by the US Department of Agriculture and the University of Washington's Rural Health Research Center, categorizing US census tracts based on population density, urbanization, and daily commuting patterns.[13]

IBD-specific medication classes included 5-aminosalicylic acid (5-ASA), corticosteroids, immunomodulators (including azathioprine, cyclosporine, mercaptopurine, methotrexate, and tacrolimus), and advanced therapies (including anti–tumor necrosis factor (anti-TNF) biologics, anti-integrin biologics, anti–interleukin (IL)-12/23 biologics, Janus kinase (JAK) inhibitors, and sphingosine 1-phosphate modulators) and were defined by RxNorm codes and measured after the index date (Supplemental Table 1).

Baseline patient characteristics were summarized, and each theme of the SVI was categorized by quartiles (0%–25%, 25%–50%, 50%–75%, 75%–100%), with the highest quartile (Q4; 75%–100%) representing the highest level of social vulnerability (either overall or in each theme, respectively). Multivariate regression was utilized to evaluate the relationship between socioeconomic factors and medication prevalence. Odds ratios (ORs) and 95% confidence intervals (CIs) were computed to assess the significance of these predictors. *P* values less than 0.05 were considered statistically significant. Data extraction was carried out using Microsoft SQL Server queries, and all statistical analyses were performed in R Studio.

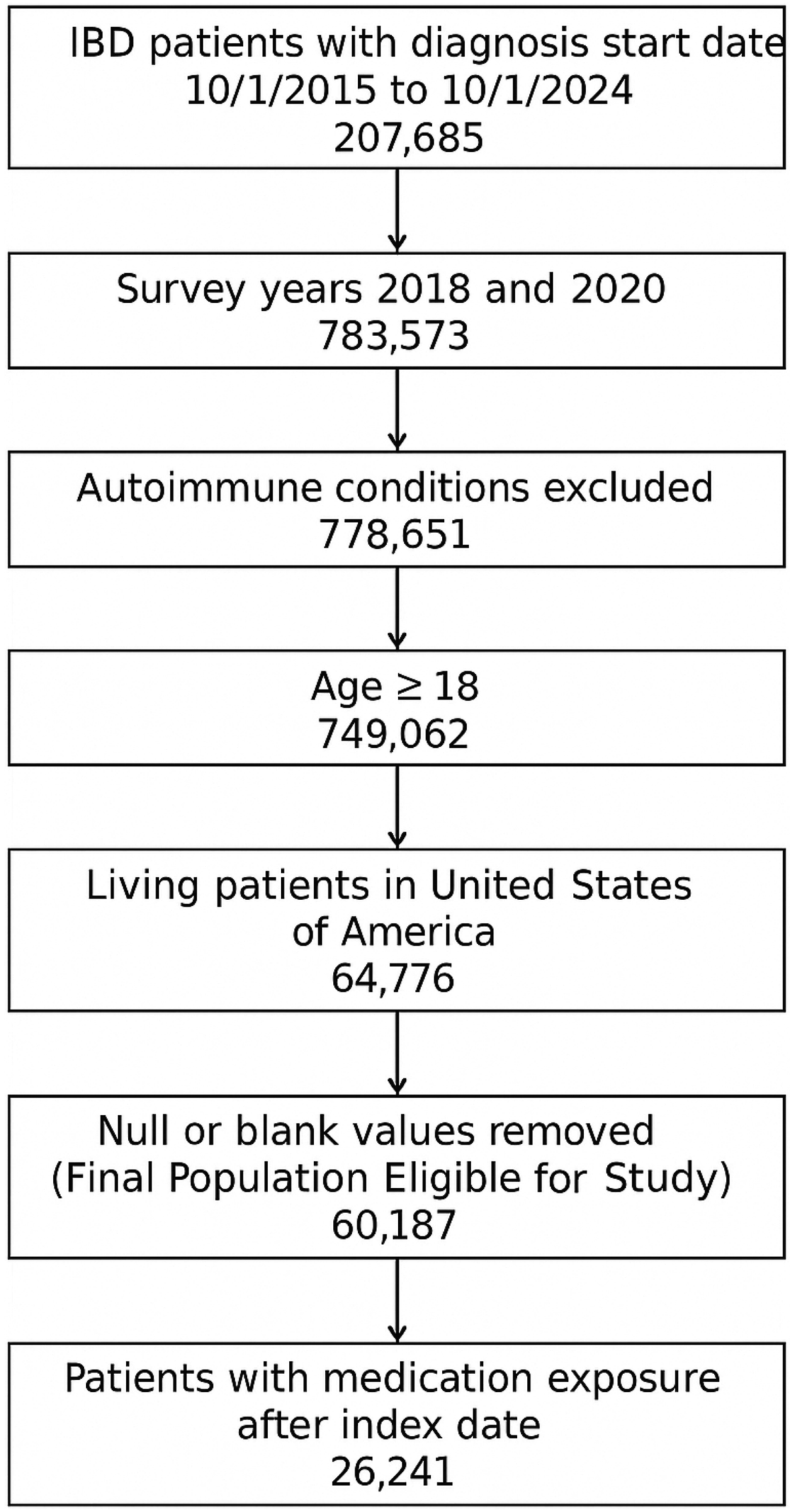

Figure 1. The flowchart of the study cohort with IBD in Epic Cosmos, 2015–2024.

Results

Cohort Demographics

A total of 60,187 patients met eligibility criteria (Table 1). The cohort was balanced by sex (57.1% women) and equally represented CD and UC. Most patients were middle-aged and older, with more than half ages >46 years old. The population was predominantly White (83.5%), with 10.5% Black, 1.8% Asian, and smaller proportions of other racial groups. A small proportion of patients (7.6%) identified as Hispanic or Latino.

Table 1. Demographic Breakdown of the Study Cohort With IBD in Epic Cosmos, 2015–2024

Demographic	Variable	N (%)
Inflammatory bowel disease (IBD) classification	Crohn's disease	30,341 (50.4%)
	Ulcerative colitis	29,846 (49.6%)
Sex	Female	34,364 (57.1%)
	Male	25,823 (42.9%)
Age	18–25	11,328 (18.8%)
	26–45	17,363 (28.8%)
	46–65	18,580 (30.9%)
	>65	12,916 (21.5%)
Race	Asian	1081 (1.8%)
	Black or African American	608 (10.1%)
	American Indian or Alaska Native	576 (1.0%)
	Native Hawaiian or other Pacific Islander	96 (0.2%)
	Other race	1663 (2.8%)
	White	50,266 (83.5%)
Ethnicity	Not Hispanic or Latino	55,607 (92.4%)
	Hispanic or Latino	4580 (7.6%)
Insurance type	Medicaid	6580 (10.9%)
	Medicare	12,122 (20.1%)
	Miscellaneous/other	37,569 (62.4%)
	Self-pay	3916 (6.5%)
Rural-Urban Commuting Area (RUCA) code	Rural	3982 (6.6%)
	Urban	56,205 (93.4%)
Overall Social-Vulnerability Index (SVI)	0%–25%	10,467 (17.4%)
	25%–50%	13,997 (23.3%)
	50%–75%	17,042 (28.3%)
	75%–100%	18,681 (31.0%)
SVI racial and ethnic minority status	0%–25%	4331 (7.2%)
	25%–50%	12,651 (21.0%)
	50%–75%	24,432 (40.6%)
	75%–100%	18,776 (31.2%)
SVI socioeconomic status	0%–25%	14,162 (23.5%)
	25%–50%	14,259 (23.7%)
	50%–75%	15,993 (26.6%)
	75%–100%	15,773 (26.2%)
SVI housing type and transportation status	0%–25%	10,543 (17.5%)
	25%–50%	13,445 (22.3%)
	50%–75%	17,430 (29.0%)
	75%–100%	18,769 (31.2%)
SVI household status	0%–25%	9803 (16.3%)
	25%–50%	14,180 (23.6%)
	50%–75%	18,941 (31.5%)
	75%–100%	17,263 (28.7%)

The majority of patients resided in urban communities (93.4%) and was insured through nongovernmental or mixed plans, while roughly one-third were covered by public insurance (Medicaid or Medicare). Social vulnerability was widely distributed across quartiles, indicating broad socioeconomic representation within the cohort. Due to limited sample size (<11 patients), data on sphingosine 1-phosphate–modulators could not be reported in accordance with Epic Cosmos privacy regulations.

Age

The reference group was patients aged 18–25 years old. In adjusted models, the likelihood of receiving advanced biologic therapy decreased steadily with age. Relative to patients aged 18–25 years, those aged 26–45, 46–65, and 65+ years were less likely to receive anti–IL-12/23 biologics (ORs 0.75, 0.65, and 0.27, respectively; all $P < .01$) and anti-TNF biologics (ORs 0.46, 0.37, and 0.15, respectively; all $P < .01$).

Use of additional IBD medication classes also declined with age. Patients aged 26–45 years were less likely than those aged 18–25 years to receive immunomodulators (OR 0.78, 95% CI 0.69–0.89; $P < .01$). JAK inhibitor use was similarly reduced among patients aged 26–45 years (OR 0.49, 95% CI 0.31–0.75) and 65+ years (OR 0.28, 95% CI 0.15–0.49; both $P < .01$). Use of 5-ASAs was also lower in ages 26–45 (OR 0.70, 95% CI 0.65–0.76; $P < .01$) and ages 46–65 years (OR 0.81, 95% CI 0.75–0.87; $P < .01$).

In contrast, corticosteroid prescription trends increased with age. Patients aged 26–45 years were less likely to receive corticosteroids (OR 0.90, 95% CI 0.85–0.95), whereas those aged 46–65 years (OR 1.13, 95% CI 1.07–1.19) and 65+ years (OR 1.14, 95% CI 1.08–1.21) were more likely ($P < .01$ for all) (Table 2).

Female Sex

Women aged 18–25 years were more likely to receive anti-TNF biologics as compared to women older than 25 years (women age 26–45 years (OR 0.76, 95% CI [0.64, 0.89], $P = .001$), 46–65 years (OR 0.46, 95% CI [0.39, 0.55], $P < .001$), and 65+ (OR 0.16, 95% CI [0.12, 0.21], $P < .001$)). Conversely, women aged 26–45 years (OR 1.19, 95% CI [1.08, 1.32], $P = .001$), 46–65 years (OR 1.40, 95% CI [1.27, 1.55], $P < .001$), and 65+ (OR 1.48, 95% CI [1.30, 1.67], $P < .001$) were significantly more likely to receive corticosteroids. Women aged 65+ were less likely to receive anti–IL-12/23 biologics (OR 0.25, 95% CI [0.16, 0.38], $P < .001$), 5-ASAs (OR 1.30, 95% CI [1.13, 1.50], $P < .001$), and JAK inhibitors (OR 0.28, 95% CI [0.09, 0.76], $P = .0.17$). Women aged 46–65 years were less likely to receive anti–IL-12/23 biologics (OR 0.61, 95% CI [0.46, 0.79], $P < .001$).

There was no notable difference between these age groups in receiving anti-integrin biologics and immunomodulators (Supplemental Table 2).

Insurance Status

Patients on Medicare were less likely than those on Medicaid to receive immunomodulators (OR 0.81, 95% CI [0.69, 0.96], $P = .016$) and 5-ASAs (OR 0.83, 95% CI [0.75, 0.92], $P < .001$). There were no notable differences between insurance type (Medicaid, Medicare, miscellaneous/other, and self-pay) and receiving JAK inhibitors, anti–IL-12/23 biologics, anti-TNF biologics, anti-integrin biologics, and corticosteroids (Supplemental Table 3).

Table 2. IBD Therapy Exposure Classified by Age

Medication class	Exposed n (%)	Age range (y)	Odds ratio	Lower 95% CI	Upper 95% CI	*P* value
Anti-TNF biologics	2095 (3.48%)	18–25	Reference	–	–	–
		26–45	0.46	0.42	0.52	**<.001**
		46–65	0.37	0.33	0.41	**<.001**
		65+	0.15	0.13	0.18	**<.001**
Anti-integrin biologics	213 (0.35%)	18–25	Reference	–	–	–
		26–45	1.12	0.77	1.65	.57
		46–65	0.95	0.65	1.42	.81
		65+	0.69	0.52	1.08	.11
Anti–IL-12/23 biologics	712 (1.18%)	18–25	Reference	–	–	–
		26–45	0.75	0.62	0.90	**.002**
		46–65	0.65	0.53	0.79	**<.001**
		65+	0.27	0.21	0.36	**<.001**
Immunomodulators	2123 (3.53%)	18–25	Reference	–	–	–
		26–45	0.78	0.69	0.89	**<.001**
		46–65	0.99	0.88	1.12	.93
		65+	0.96	0.84	1.10	**.58**
5-ASAs	5843 (9.71%)	18–25	Reference	–	–	–
		26–45	0.70	0.65	0.76	**<.001**
		46–65	0.81	0.75	0.87	**<.001**
		65+	0.99	0.92	1.07	.84
JAK inhibitors	160 (0.27%)	18–25	Reference	–	–	–
		26–45	0.49	0.31	0.75	**.001**
		46–65	0.82	0.56	1.21	.31
		65+	0.28	0.15	0.49	**<.001**
Corticosteroids	15,083 (25.1%)	18–25	Reference	–	–	–
		26–45	0.90	0.85	0.95	**<.001**
		46–65	1.13	1.07	1.19	**<.001**
		65+	01.14	1.08	1.21	**<.001**

Statistically significant values are bolded.

Stratification by Sex

There were no significant associations between men and women among all drug classes (Supplemental Table 4).

Race and Ethnicity

Asian patients were less likely to receive anti–IL-12/23 biologics than White patients (OR 0.31, 95% CI [0.10, 0.72], $P = .019$). American Indian or Alaska Native patients were less likely to receive 5-ASAs than White patients (OR 0.70, 95% CI [0.50, 0.95], $P = .029$). Race did not significantly correlate to other medication classes (Table 3). Patients that were Hispanic or Latino were more likely than those that were not to receive corticosteroids (OR 1.10, 95% CI [1.02, 1.19], $P = .016$). Hispanic or Latino status was not significantly correlated to being more or less likely to receive any other medication classes (Table 4).

Social Vulnerability Index

Compared to the least vulnerable patients with IBD (first quartile [Q1] SVI), patients in Q2 were less likely to receive immunomodulators (Q2 SVI OR 0.86, 95% CI [0.75, 0.98], $P = .026$). Other SVI comparisons were not significantly associated with exposure differences in any other medication class (Table 5). Subsequently, when SVI subclasses were examined (by socioeconomic status, racial/ethnic minority status, housing and transport type, and household status), only Q2 (second quartile SVI) socioeconomic status patients were more likely to receive 5-ASAs than Q1 socioeconomic status patients (OR 1.15, 95% CI [1.03, 1.28], $P = .010$). All other medication classes were not significantly associated with SVI subclass status.

Population Density

There were no significant urban-rural differences in medication exposure (Supplemental Table 5).

Discussion

Use of advanced therapies for IBD is associated with improved patient outcomes.[1,14–17] There are many factors impacting patient exposure to advanced therapies and this is an intense area of study. SDOH, such as age, sex, race, and socioeconomic status have emerged as factors that can greatly influence IBD therapy approach and outcomes.[5–7,18,19] The relationship between patient demographics and access to many of the newly approved advanced IBD therapies remains unclear. The datasets utilized to study IBD therapies are limited in their size, generalizability, timeliness of the data, and other factors. To our knowledge, this is the first study to examine patterns of IBD care in Epic Cosmos. Our aim was to assess the nationwide US IBD population and seek associations between therapy exposure, demographics, SDOH, and geographic characteristics in order to reveal potential health-care disparities in our IBD population. Conducting a

Table 3. IBD Medication Exposure Classified by Race

Medication class	Exposed n (%)	Race	Odds ratio	Lower 95% CI	Upper 95% CI	*P* value
Anti-TNF biologics	2095 (3.48%)	White (n = 1729)	Reference	–	–	–
		Black or AA (n = 201)	0.95	0.81	1.10	.48
		Asian (n = 40)	1.07	0.77	1.46	.67
		Other (n = 85)	1.43	1.10	1.84	**.006**
		NH or OPI (n = 2)	0.59	0.10	1.88	.47
		AI or AN (n = 20)	1.00	0.61	1.52	.99
Anti-integrin biologics	213 (0.35%)	White (n = 185)	Reference	–	–	–
		Black or AA (n = 17)	0.77	0.19	2.02	.65
		Asian (n = 3)	0.77	0.19	2.02	.65
		Other (n = 3)	0.46	0.11	1.31	.20
		NH or OPI (n = 0)	0.00	0.00	0.00	.96
		AI or AN (n = 3)	1.38	0.34	3.66	.58
Anti–IL-12/23 biologics	712 (1.18%)	White (n = 588)	Reference	–	–	–
		Black or AA (n = 88)	1.21	0.95	1.52	.11
		Asian (n = 4)	0.31	0.10	0.72	**.019**
		Other (n = 20)	1.00	0.59	1.60	.99
		NH or OPI (n = 3)	2.72	0.66	7.29	.09
		AI or AN (n = 8)	1.16	0.53	2.20	.67
Immunomodulators	2123 (3.53%)	White (n = 1767)	Reference	–	–	–
		Black or AA (n = 204)	0.97	0.83	1.12	.66
		Asian (n = 47)	1.27	0.93	1.69	.12
		Other (n = 64)	1.03	0.77	1.36	.82
		NH or OPI (n = 2)	0.58	0.10	1.84	.45
		AI or AN (n = 24)	1.18	0.76	1.74	.43
5-ASAs	5843 (9.71%)	White (n = 4918)	Reference	–	–	–
		Black or AA (n = 609)	1.02	0.93	1.12	.64
		Asian (n = 86)	0.80	0.64	0.99	.05
		Other (n = 144)	0.89	0.73	1.07	.24
		NH or OPI (n = 9)	0.96	0.45	1.80	.90
		AI or AN (n = 40)	0.70	0.50	0.95	**.029**
JAK inhibitors	160 (0.27%)	White (n = 127)	Reference	–	–	–
		Black or AA (n = 22)	1.49	0.91	2.33	.10
		Asian (n = 1)	0.36	0.02	1.61	.31
		Other (n = 6)	1.77	0.63	4.20	.23
		NH or OPI (n = 0)	0.000	0.000	0.000	.97
		AI or AN (n = 2)	1.43	0.23	4.51	.62
Corticosteroids	15,083 (25.06%)	White (n = 12,552)	Reference	–	–	–
		Black or AA (n = 1553)	1.03	0.97	1.10	.30
		Asian (n = 278)	1.04	0.91	1.20	.54
		Other (n = 417)	0.94	0.83	1.07	.36
		NH or OPI (n = 25)	1.05	0.65	1.63	.85
		AI or AN (n = 150)	1.05	0.87	1.26	.63

Statistically significant values are bolded.
AA, African American; AI, American Indian; AN, Alaska Native; NH, Native Hawaiian; OPI, Other Pacific Islander.

Table 4. IBD Medication Exposure Classified by Ethnicity

Medication class	Exposed n (%)	Ethnicity	Odds ratio	Lower 95% CI	Upper 95% CI	*P* value
Anti-TNF biologics	2095 (3.48%)	Not Hispanic or Latino (n = 1908)	Reference	–	–	–
		Hispanic or Latino (n = 187)	1.05	0.87	1.26	.59
Anti-integrin biologics	213 (0.35%)	Not Hispanic or Latino (n = 197)	Reference	–	–	–
		Hispanic or Latino (n = 16)	1.12	0.62	1.89	.68
Anti–IL-12/23 biologics	712 (1.18%)	Not Hispanic or Latino (n = 658)	Reference	–	–	–
		Hispanic or Latino (n = 54)	1.01	0.73	1.37	.95
Immunomodulators	2123 (3.53%)	Not Hispanic or Latino (n = 1946)	Reference	–	–	–
		Hispanic or Latino (n = 177)	1.11	0.93	1.33	.24
5-ASAs	5843 (9.71%)	Not Hispanic or Latino (n = 5422)	Reference	–	–	–
		Hispanic or Latino (n = 421)	0.97	0.86	1.09	.57
JAK inhibitors	160 (0.27%)	Not Hispanic or Latino (n = 149)	Reference	–	–	–
		Hispanic or Latino (n = 11)	0.76	0.35	1.48	.45
Corticosteroids	15,083 (25.06%)	Not Hispanic or Latino (n = 13,872)	Reference	–	–	–
		Hispanic or Latino (n = 1211)	1.10	1.02	1.19	**.016**

Statistically significant values are bolded.

Table 5. IBD Medication Exposure Stratified by SVI Quartile (Q4 Is the Most Vulnerable)

Medication class	Exposed n (%)	SVI (n)	Odds ratio	Lower 95% CI	Upper 95% CI	*P* value
Anti-TNF biologics	2095 (3.48%)	0%–25% (n = 387)	Reference	–	–	–
		25%–50% (n = 495)	0.95	0.83	1.09	.43
		50%–75% (n = 565)	0.89	0.78	1.02	.098
		75%–100% (n = 648)	0.93	0.81	1.06	.27
Anti-integrin biologics	213 (0.35%)	0%–25% (n = 37)	Reference	–	–	–
		25%–50% (n = 51)	1.03	0.68	1.59	.89
		50%–75% (n = 60)	1.00	0.78	1.29	.99
		75%–100% (n = 65)	1.02	0.67	1.31	.94
Anti–IL-12/23 biologics	712 (1.18%)	0%–25% (n = 130)	Reference	–	–	–
		25%–50% (n = 158)	0.90	0.71	1.14	.38
		50%–75% (n = 193)	0.90	0.72	1.13	.37
		75%–100% (n = 231)	0.97	0.77	1.21	.75
Immunomodulators	2123 (3.53%)	0%–25% (n = 394)	Reference	–	–	–
		25%–50% (n = 455)	0.86	0.75	0.98	**.026**
		50%–75% (n = 623)	0.96	0.85	1.10	.57
		75%–100% (n = 651)	0.91	0.80	1.04	.17
5-ASAs	5843 (9.71%)	0%–25% (n = 1030)	Reference	–	–	–
		25%–50% (n = 1370)	1.00	0.92	1.09	.93
		50%–75% (n = 1631)	0.97	0.90	1.06	.53
		75%–100% (n = 1812)	0.99	0.91	1.07	.78
JAK inhibitors	160 (0.27%)	0%–25% (n = 25)	Reference	–	–	–
		25%–50% (n = 44)	1.29	0.94	1.76	.32
		50%–75% (n = 45)	1.06	0.79	1.76	.81
		75%–100% (n = 46)	0.95	0.58	1.59	.83
Corticosteroids	15,083 (25.06%)	0%–25% (n = 2657)	Reference	–	–	–
		25%–50% (n = 3423)	0.95	0.90	1.01	.086
		50%–75% (n = 4274)	0.98	0.92	1.03	.43
		75%–100% (n = 4729)	0.98	0.93	1.04	.54

Statistically significant values are bolded.

retrospective study using Epic Cosmos, we gathered descriptive and demographic data on 60,187 IBD patients and performed analyses on exposure odds of IBD-specific medications. We found that certain advanced IBD therapies were less likely to be prescribed to older adults, women of traditional childbearing years (ages 26–45 years old), and certain minority races. Surprisingly, SVI and individual SVI components (socioeconomic status, racial ethnic minority, household, and housing/transportation status) were not strong predictors of advanced IBD therapy use. Additionally, Hispanic/Latino ethnicity, rural vs urban RUCA, and sex were also not strong predictors of advanced IBD therapy use.

Our data mirror national trends, showing that IBD prevalence increases with age, and further highlights age-related prescribing patterns.[20–22] Increasing age was consistently associated with a lower likelihood of receiving advanced IBD therapies. Compared with patients 18–25 years, older age groups demonstrated progressively reduced use of anti-TNF and anti–IL-12/23 biologics. Patients 26–45 years were also less likely to receive immunomodulators, JAK inhibitors, and 5-ASAs, while those 46–65 years showed reduced use of JAK inhibitors and 5-ASAs. In contrast, corticosteroid prescribing increased with age. These findings suggest that increasing age is associated with a decreased likelihood of being treated with advanced IBD therapies, potentially reflecting clinical hesitancy to initiate immunosuppressive biologic agents in older adults, concern for comorbidity burden, or differences in disease phenotype. Long-term steroids are generally avoided in the elderly population as this does not reflect the standard of care, although our data suggest otherwise, highlighting the complexities of patient preference, insurance coverage, and corticosteroid dependency and tapering. It is challenging to distinguish whether increased age leads to decreased advanced IBD therapy usage due to a milder disease course or hesitancy secondary to concerns for an increased risk profile that advanced IBD therapies may hold in the elderly population. Likely multifactorial in nature, increasing age may represent a disparity in receiving advanced IBD therapies.

There were no significant gender-based differences between men and women regarding the likelihood of receiving IBD therapies. Studies examining the prescribing of advanced IBD therapies between sexes have been performed, with a meta-analysis reporting women being less likely to use immunomodulators, anti-TNF biologics than males and more likely to use corticosteroids.[23] However, we found that when comparing women of typical childbearing ages (ages 26–45 years) to women ages 18–25 years, childbearing-aged women were less likely to receive anti-TNF biologics and more likely to receive corticosteroids. This reveals a potential hesitancy to use anti-TNF biologics in women of childbearing years, although anti-TNF biologics are traditionally viewed as a safe medication to continue throughout pregnancy.

Our study population reflects a large, nationwide IBD population, consisting of predominantly White (83.5%)

patients, matching the reported nationwide trend of a predominantly White IBD population (75%–90%), compared with Black, Hispanic, and Asian Americans.[20–22,24–26] Similarly, the Asian population has been reported to comprise 1%–2% of the IBD population[25]—our study validates such data, as our IBD population was 1.8% Asian. Studies have found a trend towards an increasing prevalence of IBD among racial minority groups.[21,25,27]

Race did not significantly correlate with most advanced IBD therapy use in our findings, except that Asian patients were less likely to receive anti–IL-12/23 biologics, and American Indian or Alaska Native patients were less likely to receive 5-ASAs than White patients (Table 3). Likewise, ethnicity did not significantly correlate with most advanced IBD therapy medication use, although Hispanic/Latino patients were more likely to receive corticosteroids than their counterparts. Previous work from our group using the OneFlorida Data Trust demonstrated decreased anti-TNF use among Black patients, while Hispanic patients showed improved access to this class of medications compared to non-Hispanic White patients.[28] Thus, it may be more helpful for a provider to view statewide or local medication trends in minority IBD populations.

Patients with Medicare were less likely to receive immunomodulators and 5-ASAs than those with Medicaid (Supplemental Table 3). It has been shown that publicly insured patients with IBD are less likely to be treated with biologics and more likely to receive corticosteroids than those with private insurance.[29]

Surprisingly, SVI and its separate subclasses (socioeconomic status, racial/ethnic minority status, housing and transport type, and household status) were not significantly associated with any medication class receival, except patients in quartile 2 (Q2 25%–50%) were less likely to receive immunomodulators than patients in Q1 (Table 5). Other advanced IBD therapies in all quartiles did not demonstrate disparities in medication receival. Rural vs urban RUCA did not significantly correlate to advanced IBD therapies (Supplementary Table 4). However, a large portion of our represented population was of an urban RUCA (93.4%), which may skew these results. These findings suggest that SVI and RUCA alone are not reliable metrics for assessing care with advanced IBD therapies. The SVI does have its own limitations, including a failure to account for community-specific variables, geographical bias (given that areas are broadly given a category that may not represent that population), and the fact that the SVI does not factor in changes over time, but rather, a single point in time.

We present up-to-date demographic data and trends regarding this population and innovatively link SVI score to receival of advanced IBD therapy. Given the complex nature of receiving advanced IBD therapies, we were unable to find a significant correlation between total SVI and most advanced IBD therapy classes. Our rigorous study design ensures chronological order of IBD diagnosis and medication use, thereby enabling a valid investigation of their association.

A major strength of this study is the use of Epic Cosmos, a nationwide EHR network encompassing more than 300 million patients in the United States. This large patient population provides great statistical power and enhances the generalizability of our findings across diverse demographic and socioeconomic populations. Unlike administrative claims databases, Epic Cosmos captures clinical data directly from EHRs, including medication prescriptions and demographic data. Using the same Epic system of individual institutions, and data harmonization, Cosmos has standardized data collection system across many institutions, minimizing variation due to inconsistent data structure. Furthermore, the integration of census-tract-derived SVI and RUCA codes within the dataset enables examination of potential social and geographic inequities in IBD care. Collectively, these features strengthen the reliability, generalizability, and relevance of our study, emphasizing Epic Cosmos as an emerging and powerful resource for population-level research in IBD.

Additionally, we performed an extensive review regarding IBD medication management as impacted by race/ethnicity.[30] One study utilized the Sinai-Helmsley Alliance for Research Excellence cohort, which pulls patients from 7 academic IBD centers. Another utilized Medicaid databases from several states.[31] This article notes that racial/ethnic disparities were not consistent across studies. One study utilized 26 million patient visits, pulling data from the National Ambulatory Medical Care Survey and the National Hospital Ambulatory Medical Care Survey from 1998 to 2010.[32] Many cited studies in this review used data from single-centers, typically academic institutions. This highlights a major advantage of our study data in that we have a robust, nationwide representative population of more than 1800 hospitals, rather than a single or even multiple institutions. These advantages position our study as a robust, population-based assessment of IBD care patterns in the modern therapeutic era.

However, despite the wide scope and detail of Epic Cosmos, several limitations must be acknowledged. Neither disease severity, a key determinant of treatment choice, was examined nor were patient comorbidities, individual physician/institutional prescribing preferences, or patient adherence to their treatment regimen. There is a lack of a systemic approach in Epic Cosmos to extract unstructured data at this time, such as imaging, which can be key to determining IBD disease severity for future research. Epic Cosmos data contribution is an opt-in, optional process, thus, not every hospital, clinic, etc. that uses the Epic EHR system contributes to the Epic Cosmos dataset, leading to selection bias. Furthermore, another source of selection bias can be attributed to the fact that patients who lack health insurance are less likely to seek health care. Although age and Medicare eligibility, and individual race/ethnicity and the SVI racial/ethnic minority subcomponent, have structural or conceptual overlap, formal collinearity testing did not indicate meaningful multicollinearity: generalized variance inflation factors for all covariates were below the threshold of concern (generalized variance inflation factor$\hat{}(1/(2\cdot \mathrm{Df})) < 1.2$ for all flagged pairs), and age-specific effect estimates were stable regardless of

insurance status inclusion (Cramér V = 0.018 for the age-insurance association). These findings support the validity of the adjusted associations reported.

Given that this study is based solely on ICD codes, medication exposure was inferred from prescriptions, rather than confirmed patient use. Another form of bias lies in that most advanced therapies for IBD are initiated and guided by a gastroenterology specialist; thus, our study does not account for those patients who did not see such a specialist and may have had limited options for IBD treatment with solely their primary or urgent care providers. Rather than a prescribing discrepancy, this reveals an inequality in subspecialty access/care. Initiation of advanced therapies for IBD is determined by the patient's disease severity as well as additional modifiers such as presence of additional autoimmune diseases, extraintestinal manifestations etc. As is the limitation with all database studies, our results do not take certain factors into account which reflect a physician's clinical decision-making process for these factors, such as imaging findings, surgical history, endoscopic findings, lab values, etc.

Additionally, our study may have limited generalizability for patients that receive health care from non-Epic systems, such as the Veterans Affairs health system. However, given that Cosmos encompasses data on approximately 300 million individuals in the United States, our study population is broadly representative of the national landscape. This study also does not capture medication switching, combination therapy analysis, or treatment response, which impact IBD management.

Conclusion

This is the first nationwide assessment of IBD care using Epic Cosmos to our knowledge. In this cohort, older adults were less likely to receive advanced IBD therapies, and differences in medication use were observed across racial groups. Among IBD patients represented in Epic Cosmos, aggregate and individual SVI components were not strongly associated with advanced therapy use. However, this finding must be interpreted with caution, as vulnerable populations with limited or no insurance coverage, reduced access to specialty care, or limited health-care engagement may be underrepresented in this dataset. Similarly, sex and urban-rural status were not strongly associated with advanced IBD therapy use in this analysis. These findings highlight the complexity of SDOH and their implications for clinical care of CD and UC. Future research should focus on exploring these vulnerable populations with IBD to reduce disparities in care.

Supplementary Materials

Material associated with this article can be found, in the online version, at https://doi.org/10.1016/j.gastha.2026.101078.

References

1. Fudman DI, McConnell RA, Ha C, et al. Modern advanced therapies for inflammatory bowel diseases: practical considerations and positioning. Clin Gastroenterol Hepatol 2025;23(3):454–468.
2. Appiah JK, Hayat U, Garg N, et al. Emerging therapies in inflammatory bowel disease: a comprehensive review. J Clin Med 2025;14:6119.
3. Vieujean S, Jairath V, Peyrin-Biroulet L, et al. Understanding the therapeutic toolkit for inflammatory bowel disease. Nat Rev Gastroenterol Hepatol 2025;22(6):371–394.
4. Yashima K, Kurumi H, Yamaguchi N, et al. Progressing advanced therapies for inflammatory bowel disease: current status including dual biologic therapy and discontinuation of biologics. Expert Rev Gastroenterol Hepatol 2025;19(3):291–310.
5. Anyane-Yeboa A, Quezada S, Rubin DT, et al. The impact of the social determinants of health on disparities in inflammatory bowel disease. Clin Gastroenterol Hepatol 2022;20(11):2427–2434.
6. Shah R, Kelley J, Amundsen T, et al. Medical and social determinants of health as predictors of adverse outcomes in patients with inflammatory bowel disease. Proc (Bayl Univ Med Cent) 2022;36(2):165.
7. Bernstein CN, Walld R, Marrie RA. Social determinants of outcomes in inflammatory bowel disease. Am J Gastroenterol 2020;115(12):2036–2046.
8. Adepoju OE, Kiaghadi A. Measuring historic and longitudinal social vulnerability in disaster-prone communities: a modification to the centers for disease control and prevention social vulnerability index (CDC-SVI). Disaster Med Public Health Prep 2023;17(1-2):e368.
9. Swaminathan SS, Medeiros FA. Socioeconomic disparities in glaucoma severity at initial diagnosis: a nationwide electronic health record cohort analysis. Am J Ophthalmol 2024;263:50–60.
10. Vance GR, Bloodworth CS, Gleason PE, et al. Increased prevalence of psychiatric conditions in panniculectomy patients with prior bariatric surgery: a nationwide epic cosmos study. Cureus 2025;17(1):e77321.
11. Bin Noor N, Pro G, Acharya M, et al. Association between different modalities of opioid use disorder-related care delivery and opioid use disorder-related patient outcomes: a retrospective cohort study. Addict Behaviors Rep 2025;21:100588.
12. Handley SC, Gallagher K, Breden A, et al. Birth Hospital length of stay and rehospitalization during COVID-19. Pediatrics 2022;149(1):e2021053498.
13. Rural-Urban Commuting Area Codes - Documentation | Economic Research Service. https://www.ers.usda.gov/data-products/rural-urban-commuting-area-codes/documentation. Accessed April 3, 2025.
14. Kobayashi T, Hibi T. Improving IBD outcomes in the era of many treatment options. Nat Rev Gastroenterol Hepatol 2023;20(2):79–80.
15. Kobayashi T, Siegmund B, Le Berre C, et al. Ulcerative colitis. Nat Rev Dis Primers 2020;6(1):74.
16. Vuyyuru SK, Nguyen TM, Murad MH, et al. Comparative efficacy of advanced therapies for achieving endoscopic outcomes in Crohn's disease: a systematic review and

network meta-analysis. Clin Gastroenterol Hepatol 2024;22(6):1190–1199.e15.
17. Kapizioni C, Desoki R, Lam D, et al. Biologic therapy for inflammatory bowel disease: Real-World comparative effectiveness and impact of drug sequencing in 13 222 patients within the UK IBD BioResource. J Crohns Colitis 2024;18(6):790–800.
18. Piovani D, Danese S, Peyrin-Biroulet L, et al. Environmental, nutritional, and socioeconomic determinants of IBD incidence: a global ecological study. J Crohns Colitis 2020;14(3):323–331.
19. Sewell JL, Velayos FS. Systematic review: the role of race and socioeconomic factors on IBD healthcare delivery and effectiveness. Inflamm Bowel Dis 2013; 19(3):627.
20. Lewis JD, Parlett LE, Jonsson Funk ML, et al. Incidence, prevalence, and racial and ethnic distribution of inflammatory bowel disease in the United States. Gastroenterology 2023;165(5):1197–1205.e2.
21. Xu F, Dahlhamer JM, Zammitti EP, et al. Health-risk behaviors and chronic conditions among adults with inflammatory bowel disease — united States, 2015 and 2016. MMWR Morb Mortal Wkly Rep 2019; 67(6):190–195.
22. Ye Y, Manne S, Treem WR, et al. Prevalence of inflammatory bowel disease in pediatric and adult populations: recent estimates from large national databases in the United States, 2007-2016. Inflamm Bowel Dis 2020; 26(4):619–625.
23. Shah YR, Sebastian SA, Dahiya DS, et al. Sex-based disparities in treatment and healthcare utilization in patients with ulcerative colitis: a systematic review and meta-analysis. J Clin Med 2024;13(24):7534.
24. Alchirazi KA, Mohammed A, Eltelbany A, et al. S1045 racial disparities in utilization of medications in inflammatory bowel disease patients. Am J Gastroenterol 2022;117(10S):e758–e759.
25. Barnes EL, Nowell WB, Venkatachalam S, et al. Racial and ethnic distribution of inflammatory bowel disease in the United States. Inflamm Bowel Dis 2022; 28(7):983–987.
26. Cai Q, Ding Z, Fu AZ, et al. Racial or ethnic differences on treatment adherence and persistence among patients with inflammatory bowel diseases initiated with biologic therapies. BMC Gastroenterol 2022;22(1):1–12.
27. Afzali A, Cross RK. Racial and ethnic minorities with inflammatory bowel disease in the United States: a systematic review of disease characteristics and differences. Inflamm Bowel Dis 2016;22(8):2023–2040.
28. Hussain FS, Potlach T, Chi X, et al. Healthcare utilization and geographic distribution of advanced therapy in minority race and ethnic groups with inflammatory bowel disease. Inflamm Bowel Dis 2025;31(2):344–350.
29. Potlach T, Zhang Y, Naueen C, et al. Sa1018 publicly insured patients with IBD, including those with severe disease, are more likely to receive steroids and less likely to be treated with advanced therapies than privately insured patients. Gastroenterology 2024;166(5):S-321.
30. Barnes EL, Loftus EV, Kappelman MD. Effects of race and ethnicity on diagnosis and management of inflammatory bowel diseases. Gastroenterology 2021;160(3):677–689.
31. Flasar M, Mulani PM, Yang M, et al. Racial differences in use of biologics for crohn's disease in a medicaid population. Gut 2011;60(Suppl 1):A138.
32. Lin KK, Sewell JL. The effects of race and socioeconomic status on immunomodulator and anti-tumor necrosis factor use among ambulatory patients with inflammatory bowel disease in the United States. Am J Gastroenterol 2013;108(12):1824–1830.

Received February 9, 2026. Accepted July 27, 2026.

Correspondence:
Address correspondence to: Hannah Zuercher, MD, Division of Gastroenterology and Hepatology, Department of Internal Medicine, University of Florida, 7596 SW 90thRd, Gainesville, Florida 32608. e-mail: hannah.safadi@utah.edu.

Acknowledgments:
We are grateful for the support of the University of Florida College of Medicine, as well as our research team members for their contributions to enhance the quality of this study.

Authors' Contributions:
Hannah Zuercher and Tomas Potlach were involved in conception/study design, interpretation of data, and manuscript drafting/revision. Mousumi Sinha was involved in data generation/collection and analysis. Shay S. Bidani and Sean Olson were involved in manuscript revision. Naveen Baskaran, Tianze Jiao, Ellen M. Zimmermann, and Naueen A. Chaudhry were involved in study design, manuscript revision, and final manuscript approval. All authors have made substantial contributions to all of the following: the conception and design of the study, acquisition of data, analysis and interpretation of data, drafting the article or revising it critically for important intellectual content, or final approval of the version to be submitted.

Conflicts of Interest:
The authors disclose no conflicts.

Funding:
This research did not receive any specific grant from funding agencies in the public, commercial, or not-for-profit sectors. The University of Florida Inflammatory Bowel and Celiac Disease Program receives support from the Bishop Parker Foundation.

Ethical Statement:
Institutional review board approval was not required for this data collection and publication, given that data were obtained deidentified.

Data Transparency Statement:
Data, analytic methods, and study material will be made available to other researchers in the supplementary section.

Reporting Guidelines:
RECORD.

Gastro Hep Advances, Volume 5

Supplemental information

Age, Rather Than Social Vulnerability, Is Associated With Disparities in Prescription Patterns in Inflammatory Bowel Disease: An Epic Cosmos Study

Hannah Zuercher, Tomas Potlach, Mousumi Sinha, Naveen Baskaran, Shay S. Bidani, Sean Olson, Tianze Jiao, Ellen M. Zimmermann, and Naueen A. Chaudhry

Supplemental Table 1: List of RxNorm Codes Used for Identification of Medication Classes.

Medication Class	Medications	RxNorm
Anti-TNF Biologics	Adalimumab	327361
	Certolizumab	709271
	Golimumab	819300
	Infliximab	191831
Anti-Integrin Biologics	Natalizumab	354770
	Vedolizumab	1538097
Anti-IL-12/23 Biologics	Ustekinumab	847083
	Risankizumab	2166040
	Mirikizumab	2668384
Immunomodulators	Azathioprine	1256
	Cyclosporine	3008
	Mercaptopurine	103
	Methotrexate	6851
	Tacrolimus	42316
5-ASAs	Balsalazide	18747
	Mesalamine	52582
	Sulfasalazine	9524
JAK Inhibitors	Tofacitinib	1357536
	Upadacitinib	2196092
S1P Modulators	Ozanimod	2288236
	Etrasimod	2668045
Corticosteroids	Budesonide	19831
	Methylprednisolone	6902
	Prednisone	8640

Abbreviations: TNF - Tumor Necrosis Factor, IL - Interleukin, ASA - Aminosalicylic Acid, JAK - Janus Kinase, S1P - Sphingosine 1-phosphate

Supplemental Table 2: IBD Medication Exposure Classified by Female Age Group (Reference Group is Age 18-25 years old).

Medication Class	Age Group (Years)	Odds Ratio	Lower 95% CI	Upper 95% CI	*P*-value
Anti-TNF Biologics	26-45	0.76	0.64	0.89	**< .001**
	46-65	0.46	0.39	0.55	**< .001**
	65+	0.16	0.12	0.21	**< .001**
Anti-Integrin	26-45	1.06	0.64	1.77	.822
	46-65	0.81	0.48	1.37	.415
	65+	0.62	0.30	1.25	.190
Anti-IL-12/23	26-45	0.91	0.71	1.17	.469
	46-65	0.61	0.46	0.79	**< .001**
	65+	0.25	0.16	0.38	**< .001**
Immunomodulators	26-45	0.98	0.82	1.19	.866
	46-65	1.09	0.91	1.30	.357
	65+	1.04	0.84	1.29	.741
5-ASA	26-45	0.91	0.80	1.03	.125
	46-65	0.90	0.79	1.01	.077
	65+	1.30	1.13	1.50	**< .001**
JAK Inhibitors	26-45	0.79	0.40	1.57	.494
	46-65	1.08	0.59	2.07	.803
	65+	0.28	0.09	0.76	**.017**
Corticosteroids	26-45	1.19	1.08	1.32	**.001**
	46-65	1.40	1.27	1.55	**< .001**
	65+	1.48	1.30	1.67	**< .001**

Abbreviations: TNF - Tumor Necrosis Factor, IL - Interleukin, ASA - Aminosalicylic Acid, JAK - Janus Kinase

Supplemental Table 3: IBD Medication Exposure Classified by Insurance Status.

Medication Class	Exposed n (%)	Insurance Status	Odds Ratio	Lower 95% CI	Upper 95% CI	*P*-value
Anti-TNF Biologics	2,095 (3.48%)	Medicaid (n= 225)	Reference	–	–	–
		Medicare (n= 369)	0.95	0.80	1.12	.528
		Miscellaneous/Other (n=1,372)	1.09	0.94	1.26	.258
		Self-Pay (n= 129)	0.96	0.76	1.19	.693
Anti-Integrin Biologics	213 (0.35%)	Medicaid (n= 16)	Reference	–	–	–
		Medicare (n= 47)	1.56	1.10	2.22	.128
		Miscellaneous/Other (n=132)	1.44	0.88	2.53	.168
		Self-Pay (n= 18)	1.93	1.12	3.31	.057
Anti-IL-12/23 Biologics	712 (1.18%)	Medicaid (n= 75)	Reference	–	–	–
		Medicare (n= 157)	1.20	0.91	1.60	.199
		Miscellaneous/Other (n=433)	1.05	0.82	1.35	.717
		Self-Pay (n=47)	1.07	0.73	1.54	.727
Immunomodulators	2,123 (3.53%)	Medicaid (n= 239)	Reference	–	–	–
		Medicare (n= 359)	0.81	0.69	0.96	**.016**
		Miscellaneous/Other (n=1,363)	1.00	0.87	1.16	.99
		Self-Pay (n=162)	1.15	0.94	1.41	.184
5-ASAs	5,843 (9.71%)	Medicaid (n= 690)	Reference	–	–	–
		Medicare (n= 1079)	0.83	0.75	0.92	**< .001**
		Miscellaneous/Other (n=3,678)	0.92	0.84	1.00	.062
		Self-Pay (n= 396)	0.96	0.84	1.09	.523
JAK Inhibitors	160 (0.27%)	Medicaid (n= 16)	Reference	–	–	–
		Medicare (n=33)	1.18	0.65	2.21	.593
		Miscellaneous/Other (n=98)	1.11	0.67	1.56	.71
		Self-Pay (n=13)	1.36	0.64	2.10	.413
Corticosteroids	15,083 (25.06%)	Medicaid (n= 1621)	Reference	–	–	–
		Medicare (n= 2823)	0.94	0.87	1.00	.066
		Miscellaneous/Other (n=9,611)	1.06	0.99	1.12	.073
		Self-Pay (n=1,028)	1.09	0.99	1.19	.067

Abbreviations: IBD - Inflammatory Bowel Disease, SVI - Social Vulnerability Index, CI - Confidence Interval, TNF - Tumor Necrosis Factor, IL - Interleukin, ASA - Aminosalicylic Acid, JAK - Janus Kinase

Supplemental Table 4. IBD Medication Exposure Classified by Sex.

Medication Class	Exposed n (%)	Sex	Odds Ratio	Lower 95% CI	Upper 95% CI	*P*-value
Anti-TNF Biologics	2,095 (3.48%)	Men (n=933)	1.06	0.97	1.16	.17
		Women (n=1,162)	-	-	-	-
Anti-Integrin Biologics	213 (0.35%)	Men (n=82)	0.83	0.63	1.10	.20
		Women (n=131)	-	-	-	-
Anti-IL-12/23 Biologics	712 (1.18%)	Men (n=298)	0.96	0.83	1.12	.62
		Women (n=414)	-	-	-	-
Immunomodulator s	2,123 (3.53%)	Men (n=903)	0.98	0.90	1.07	.66
		Women (n=1,220)	-	-	-	-
5-ASAs	5,843 (9.71%)	Men (n=2,496)	0.99	0.94	1.05	.85
		Women (n=3,347)	-	-	-	-
JAK Inhibitors	160 (0.27%)	Men (n=69)	1.01	0.74	1.38	.96
		Women (n=91)	-	-	-	-
Corticosteroids	15,083 (25.06%)	Men (n=6,553)	1.03	0.99	1.07	.13
		Women (n=8,530)	-	-	-	-

Abbreviations: IBD - Inflammatory Bowel Disease, SVI - Social Vulnerability Index, CI - Confidence Interval, TNF - Tumor Necrosis Factor, IL - Interleukin, ASA - Aminosalicylic Acid, JAK - Janus Kinase

Supplemental Table 5: IBD Medication Exposure Based on Rural-Urban Differences.

Medication Class	Exposed n (%)	RUCA (n)	Odds Ratio	Lower 95% CI	Upper 95% CI	*P*-value
Anti-TNF Biologics	2,095 (3.48%)	Rural (n= 128)	Reference	–	–	–
		Urban (n= 1967)	1.05	0.87	1.26	.632
Anti-Integrin Biologics	213 (0.35%)	Rural (n= 16)	Reference	–	–	–
		Urban (n=	0.89	0.55	1.54	.649

		197)				
Anti-IL-12/23 Biologics	712 (1.18%)	Rural (n= 40)	Reference	–	–	–
		Urban (n= 672)	1.18	0.87	1.66	.308
Immunomodulators	2,123 (3.53%)	Rural (n= 155)	Reference	–	–	–
		Urban (n= 1968)	0.88	0.74	1.04	.123
5-ASAs	5,843 (9.71%)	Rural (n= 365)	Reference	–	–	–
		Urban (n=5478)	1.07	0.96	1.20	.243
JAK Inhibitors	160 (0.27%)	Rural (n= 12)	Reference	–	–	–
		Urban (n= 148)	0.86	0.49	1.64	.608
Corticosteroids	15,083 (25.06%)	Rural (n= 1001)	Reference	–	–	–
		Urban (n= 14082)	0.98	0.91	1.06	.593

Abbreviations: IBD - Inflammatory Bowel Disease, SVI - Social Vulnerability Index, CI - Confidence Interval, TNF - Tumor Necrosis Factor, IL - Interleukin, ASA - Aminosalicylic Acid, JAK - Janus Kinase
